# Cervical Cancer Screening Using Papanicolaou Smear and Visual Inspection With Acetic Acid and Their Correlation With Colposcopy

**DOI:** 10.7759/cureus.88679

**Published:** 2025-07-24

**Authors:** Eva Mishra, Ekta Chaudhary, Shweta Mishra, Vishi Rawat, Shambhavi Tripathi, Richa Rathoria, Rupali Gupta, Ekansh Rathoria

**Affiliations:** 1 Obstetrics and Gynecology, Hind Institute of Medical Sciences, Sitapur, IND; 2 Pathology, Hind Institute of Medical Sciences, Sitapur, IND; 3 Pediatrics, Hind Institute of Medical Sciences, Sitapur, IND

**Keywords:** carcinoma, cervical cancer, colposcopy, mass screening, papanicolaou test, papillomavirus infections, uterine cervical neoplasms, vaginal discharge, visual inspection with acetic acid

## Abstract

Introduction: Cervical cancer remains a leading cause of morbidity and mortality among women, particularly in low- and middle-income countries (LMICs), despite being largely preventable. In India, delayed diagnosis and limited access to screening contribute to late-stage presentation. This study evaluates the effectiveness of visual inspection with acetic acid (VIA) and Papanicolaou (Pap) smear, and their correlation with colposcopic findings.

Methods: This prospective observational study was conducted over 18 months on 170 women aged 25-65 years. All participants underwent VIA and Pap smear screening. Those with abnormal findings underwent colposcopy and Swede scoring, and biopsies were taken based on a colposcopic Swede score ≥ 5. Histopathological examination (HPE) served as the gold standard for diagnosis. Statistical analysis included descriptive statistics and chi-squared testing. Diagnostic parameters, i.e., sensitivity, specificity, positive predictive value (PPV), negative predictive value (NPV), and accuracy, were calculated. A p-value < 0.05 was considered statistically significant.

Results: The mean age of participants was 40.15±8.99 years. The most common complaint was vaginal discharge in 155/170 (91.2%). VIA was positive in 56/170 (32.9%) participants, and Pap smear abnormalities were noted in 39/170 (22.9%). Among the 58 women who underwent colposcopy, cervical intraepithelial neoplasia (CIN) 1 was the most common lesion in 28/58 (48.3%), followed by chronic cervicitis in 17/58 (29.3%), and CIN 2 and CIN 3 in 5/58 (8.6%) each. Histopathology confirmed CIN 2 in 5/10 (50%), CIN 3 in 4/10 (40%), and squamous cell carcinoma (SCC) in 1/10 (10%) cases among the 10 biopsied patients. However, the histopathological validation is limited by the small number of biopsies (n=10), which was considered when interpreting diagnostic accuracy. Diagnostic performance showed that VIA had a sensitivity of 100%, specificity of 10%, PPV of 67.9%, and NPV of 100%, while Pap smear had a sensitivity of 94.7%, specificity of 85%, PPV of 92.3%, and NPV of 89.5%. Statistically significant associations were observed between VIA results and colposcopy findings (p<0.0001), Pap results and colposcopy findings (p<0.0001), and colposcopic and histopathological findings (p=0.007). The mean age at marriage of the study participants was 20.77±1.93 years. Significant associations were observed between abnormal VIA and Pap smear results with early age at marriage between 18 and 20 years (p=0.002 and p=0.008, respectively), lower socioeconomic status (p=0.001 and p=0.024, respectively), and high parity (p<0.0001 and p=0.002, respectively).

Conclusion: VIA and Pap smear serve as effective frontline screening tools, with Pap smear offering higher specificity. VIA's high sensitivity and ease of implementation make it suitable for mass screening in low-resource settings, while Pap smear provides greater diagnostic accuracy when infrastructure allows. Integrating VIA and Pap screening, followed by colposcopic confirmation, can improve early detection and reduce cervical cancer burden in LMICs. Adopting a tiered screening model, beginning with VIA at primary care levels and referring positive cases for Pap smear and colposcopy, could enhance early detection and optimize resource allocation in low-resource settings. Implementation of structured national screening programs alongside HPV vaccination is crucial to meet the WHO's 2030 cervical cancer elimination targets.

## Introduction

Cervical cancer is a malignant tumor arising from the epithelial cells of the cervix, the lowest segment of the uterus that attaches to the vagina [1]. The cervix consists of two anatomical regions: the ectocervix, lined by squamous epithelium, and the endocervix, lined by columnar epithelium [2]. The junction between these two zones, referred to as the squamocolumnar junction or transformation zone, is particularly vulnerable to oncogenic human papillomavirus (HPV) infection due to active metaplastic changes occurring in this region [2].

Globally, cervical cancer ranks as the fourth most common cancer among women, with an estimated 660,000 new cases and around 350,000 deaths in 2022, according to the World Health Organization (WHO) [3]. Despite being largely preventable, cervical cancer remains a leading cause of cancer-related mortality among women, particularly in low- and middle-income countries (LMICs), where nearly 90% of cervical cancer deaths occur [4]. This variation is due to the dearth of organized screening programs, limited healthcare infrastructure, and inadequate access to HPV vaccination in LMICs [5]. Various demographic parameters, such as high parity, early marriage, and lower socioeconomic status, are positively associated with increased risk of cervical cancer [6-8].

The histopathological subtypes of cervical cancer primarily include squamous cell carcinoma (SCC), accounting for approximately 75%-90% of cases, and adenocarcinoma (ADC), which constitutes about 10%-25% [9]. SCC typically arises in the transformation zone, while ADC originates from glandular cells of the endocervix [10]. Precise histological classification is essential for determining the prognosis, guiding staging, and formulating an effective treatment plan for cervical cancer [11].

Cervical cancer screening enables early detection of precancerous lesions, facilitating timely intervention and improved survival outcomes [11]. The commonly employed screening tools include the Papanicolaou (Pap) smear, visual inspection with acetic acid (VIA), and colposcopy, each with unique advantages and limitations [11]. The Pap smear, introduced in the 1940s, remains a cornerstone of cytological screening, where cervical epithelial cells are examined microscopically to detect dysplasia and carcinoma [11]. When combined with HPV DNA testing, its sensitivity significantly improves; however, it requires trained cytopathologists and well-equipped laboratories, and may involve delays in reporting results, limiting its use in resource-constrained settings [12].

In contrast, VIA is a low-cost, rapid screening alternative especially suitable for LMICs [13]. It involves applying 3%-5% acetic acid to the cervix and observing for acetowhite areas indicative of abnormal epithelium [13]. VIA offers immediate results and minimal infrastructure requirements, and has been endorsed by the WHO as an effective interim solution where cytology-based programs are not feasible [13]. However, VIA's sensitivity is highly dependent on the examiner's skill and may miss endocervical lesions or subtle dysplastic changes [13].

Colposcopy provides a diagnostic follow-up to abnormal Pap smear or VIA results [14]. It allows magnified visualization of cervical lesions, often enhanced with acetic acid or Lugol's iodine, and facilitates targeted biopsy for histopathological confirmation [14]. Despite its diagnostic value, colposcopy requires trained specialists and advanced equipment, which may not be widely available in underserved settings [14].

Given the preventable nature of cervical cancer through early detection and the prolonged pre-invasive phase of disease progression, implementing effective screening strategies is vital. Lack of structured screening programs is a major factor contributing to late-stage presentation and high mortality in many LMICs [15]. Therefore, integrating Pap smear and VIA with colposcopic confirmation may offer a practical and effective approach to cervical cancer screening. This study aimed to evaluate the diagnostic performance of VIA and Pap smear in cervical cancer screening and assess their correlation with colposcopic findings and associated demographic factors. We hypothesized that integrating these tools could enhance early detection of cervical neoplasia in resource-limited settings.

## Materials and methods

This prospective observational study was conducted in the Obstetrics and Gynecology Department at Hind Institute of Medical Sciences, Ataria, Sitapur, Uttar Pradesh, over 18 months from August 2023 to January 2025. Ethical approval was obtained from the Institutional Ethics Committee (approval number: lHEC-HIMSA/MD-MS-22), and written informed consent was obtained from all participants.

The study included married women aged 25-65 years attending the outpatient and inpatient Gynecology Departments with abnormal symptoms of excessive discharge, itching, bleeding after intercourse, or bleeding between periods. We excluded women with known cases of frank invasive cervical cancer, those with a history of previous surgery such as cryosurgery and hysterectomy, pregnant women, women in the three-month post-natal period, those with recent vaginal douching or pessary use within 24 hours, those with a history of sexual intercourse within 48 hours, and those receiving prior treatment for positive Pap smear/VIA or cervical intraepithelial neoplasia (CIN).

The sample size (n) was calculated using Cochrane's formula (n = z^2^.p.q/e^2^), where z is 1.96 (for 95% confidence interval), p was 0.127 based on a reported 12.7% prevalence of cervical lesions from previous study, q i.e., 1-p was 0.873, with a margin of error (e) of 0.05 [16,17]. As the exact size of the eligible population was not known, Cochran's formula was applied, assuming a large population without a finite population correction. The calculated sample size was 170.

Eligible participants were selected through simple random sampling. A detailed history, including demographic data, i.e., age, age at marriage, socioeconomic status, livelihood, and parity, and clinical data, such as chief complaints, along with their duration and menstrual, gynecological, obstetric, and contraceptive history, and past medical and surgical history, was recorded using a pre-validated proforma. A detailed general and systemic examination was performed, followed by an internal examination, which included both per speculum and per vaginal examinations. Each participant underwent a Pap smear and visual inspection with acetic acid (VIA) examination.

Pap smear procedure

After placing the patient in the lithotomy position, a sterile bivalve speculum was inserted to visualize the cervix. The ectocervical sample was collected using an Ayre spatula rotated 360°, and the endocervical sample was obtained with a cytobrush rotated 360°. The collected sample was immediately smeared on a labeled glass slide, fixed with 95% ethyl alcohol, and sent for cytological examination. Results were reported according to the new Bethesda System for Reporting Cervical Cytology 2014 [18]. The system broadly divided lesions into those negative for intraepithelial neoplasia and epithelial cell abnormalities (ECA) that included squamous and glandular cells [18]. Squamous cell abnormalities include atypical squamous cells of undetermined significance (ASCUS), atypical squamous cells but cannot rule out high-grade squamous intraepithelial lesion (HSIL) (ASC-H), low-grade squamous intraepithelial lesion (LSIL), high-grade squamous intraepithelial lesion, and squamous cell carcinoma (SCC) [18]. Glandular cell abnormalities include atypical, endocervical adenocarcinoma in situ, and adenocarcinoma [18].

Visual inspection with acetic acid (VIA)

VIA was conducted by trained gynecology residents under consultant supervision. We applied a 5% acetic acid solution to the cervix. After one minute, the cervix was inspected under adequate light. The appearance of acetowhite areas was considered VIA positive, indicating possible precancerous changes [19].

Colposcopy

Women with abnormal Pap smear or positive VIA results were referred for colposcopic evaluation using a binocular colposcope (magnification: 5×, 10×, and 20×) with a green filter for vascular pattern assessment. Swede score, an open-access validated colposcopic scoring system, was used for scoring lesions based on five criteria: uptake of acetic acid, margins and surface, vessels, lesion size, and iodine staining [20,21]. Scores of 0-4, 5-6, and 7-10 indicated normal/low-grade lesion (CIN 1 or less), possible high-grade lesion (CIN 2), and high-grade lesion/CIN 3 or invasive cancer, respectively [20].

Biopsy and histopathology

Colposcopy-guided biopsies were limited to patients with a Swede score ≥ 5, following standard protocol to reduce unnecessary invasive procedures in low-grade lesions, which may have restricted histological confirmation. It was sent in 10% formalin for histopathological examination. Tissue was processed, stained with hematoxylin and eosin, and examined under light microscopy. Findings were classified as chronic cervicitis, CIN 1, CIN 2, CIN 3, carcinoma in situ, squamous cell carcinoma, or adenocarcinoma, following WHO classification standards [22]. All patients diagnosed with CIN 2, CIN 3, or SCC were appropriately counselled and referred to our institute's higher-level gynecological oncology unit for further management, including loop excision or oncology referral where indicated.

Data collection and statistical analysis

All data were coded and entered in Microsoft Excel 2019 (Microsoft Corp., Redmond, WA). Statistical analysis was performed using IBM SPSS version 27.0 (IBM Corp., Armonk, NY). Descriptive statistics were used to summarize demographic variables and clinical findings. Continuous and categorical data were presented as mean ± standard deviation (SD) and frequency/percentage, respectively. The diagnostic test performance (sensitivity, specificity, positive predictive value (PPV), negative predictive value (NPV), and accuracy) of the screening tests was calculated. The chi-squared test was applied to examine associations between screening methods, VIA and Pap outcomes with colposcopy outcomes, and demographic characteristics. Pearson's correlation coefficient was used to assess the strength and direction of linear relationships between VIA and colposcopy, Pap smear and colposcopy, and colposcopy and histopathological outcomes. A p-value < 0.05 was considered statistically significant.

## Results

The study included 170 women between the ages of 25 and 65 years who met the inclusion and exclusion criteria. The baseline demographic characteristics of the study population are presented in Table 1.

**Table 1:** Distribution of study population according to demographics (N=170)

Demographic parameters		Number	Percentage (%)
Age group (years)	25-35	59	34.7
	36-45	54	31.8
	46-55	55	32.3
	56-65	2	1.2
Age at marriage (years)	18-20	77	45.3
	21-24	87	51.2
	≥25	6	3.5
Socioeconomic status	Upper	16	9.4
	Upper middle	19	11.2
	Lower middle	13	7.7
	Upper lower	31	18.2
	Lower	91	53.5
Livelihood	Rural	140	82.4
	Urban	30	17.6
Parity	1	39	22.9
	2	68	40
	3	51	30
	≥4	12	7.1

The mean age of study participants was 40.15±8.99 years, with 59/170 (34.7%) aged 25-35 years, while the mean age at marriage was 20.77±1.93 years, with 87/170 (51.2%) marrying between 21 and 24 years. Over half of the study population (91/170 (53.5%)) belonged to the lower socioeconomic class, most women (140/170 (82.4%)) came from rural areas, and the majority (131/170 (77.1%)) had two or more children (Table 1).

The clinical parameters of the study population are presented in Table 2, which highlights that while a majority presented with symptoms suggestive of cervical pathology, many had normal clinical findings on examination, underscoring the need for routine screening.

**Table 2:** Distribution of study population according to clinical parameters (N=170)

Clinical parameters		Number	Percentage (%)
Chief complaints	White/blood/curdy discharge ± itching	155	91.2
	Postmenopausal bleeding	11	6.4
	Intermenstrual bleeding	2	1.2
	Postcoital bleeding	2	1.2
Per speculum findings	Normal-looking cervix	84	49.4
	Hypertrophied cervix ± erosion/nabothian cyst	78	45.9
	Hypertrophied cervix + discharge	3	1.8
	Normal-looking cervix + discharge	5	2.9
Per vaginal findings	Normal	134	78.8
	Uterus normal, tenderness	23	13.5
	Bulky uterus, bleeds on touch, tender	13	7.7

Screening with VIA and Pap was conducted on all 170 study participants. VIA was positive in 56/170 (32.9%), and abnormal Pap results were found in 39/170 (22.9%). Among the 39 patients with abnormal Pap results, 37/39 (94.9%) were also VIA positive and 2/39 (5.1%) were VIA negative. A total of 58 patients with abnormal Pap smear results and positive VIA findings underwent further evaluation through colposcopy. Colposcopy revealed that 48/58 (82.8%) participants had Swede scores between 0 and 4, suggestive of normal or low-grade lesions (CIN 1 or less). Swede scores between 5 and 6 were seen in 5/58 (8.6%) patients, indicating possible high-grade changes (CIN 2), and 5/58 (8.6%) patients had scores ≥ 7, suggestive of CIN 3 or invasive lesions. Colposcopy-guided biopsy was performed in only 10/58 (17.2%) patients who had a Swede score of 5 or more identified during colposcopy. The screening tests and confirmatory test investigations of the study population are presented in Table 3. Varying degrees of epithelial abnormalities on Pap cytology (atypical glandular cells of undetermined significance (ASCUS), LSIL, HSIL, and AGUS) were seen in 39/170 (22.9%) patients. Among 131 women who were negative for intraepithelial lesion or malignancy (NILM) on Pap, 2/131 (1.5%) had fungal hyphae, 3/131 (2.3%) had bacterial vaginosis, and 6/131 (4.6%) had an inflammatory smear, while the rest of 120/131 (91.6%) were normal. Among those 10 biopsied, CIN 2 reported in 5/10 (50%) and CIN 3 in 4/10 (40%) were the most prevalent (Table 3).

**Table 3:** Distribution of the study population according to investigation results (N=170) VIA: visual inspection of acetic acid, Pap: Papanicolaou smear, HPE: histopathological examination, AGUS: atypical glandular cells of undetermined significance, ASCUS: atypical squamous cells of undetermined significance, HSIL: high-grade squamous intraepithelial lesion, LSIL: low-grade squamous intraepithelial lesion, NILM: negative for intraepithelial lesion or malignancy, CIN: cervical intraepithelial neoplasia, SCC: squamous cell carcinoma

Investigations			Number	Percentage (%)
VIA results	Negative		114	67.1
	Positive		56	32.9
Pap results	NILM ± (inflammatory/fungal hyphae/bacterial vaginosis)		131	77.1
	ASCUS		24	14.1
	AGUS		2	1.2
	LSIL		9	5.3
	HSIL		4	2.3
Colposcopy findings	Not conducted		112	65.9
	Normal		3	1.8
	Chronic cervicitis		17	10
	CIN 1		28	16.5
	CIN 2		5	2.9
	CIN 3		5	2.9
HPE results	Swede score < 5	Not conducted	160	94.1
	Swede score ≥ 5	CIN 2	5	2.9
		CIN 3	4	2.4
		SCC	1	0.6

A statistically significant association was observed between the outcomes of both VIA and Pap smear and the corresponding colposcopic findings (p<0.0001) (Table 4). All VIA-positive cases were linked with abnormal colposcopic findings (CIN 1-3 or chronic cervicitis), while VIA-negative results were mostly associated with normal findings. Although VIA was significantly associated with colposcopic findings, the weak, non-significant correlation (Pearson's R=-0.177, p=0.183) reflects its binary nature and supports its use as a screening tool rather than for grading lesion severity. However, Pap smear abnormalities (AGUS, ASCUS, LSIL, and HSIL) strongly correlated with the severity of colposcopic lesions (Pearson's R=0.681, p<0.0001). Most ASCUS and LSIL cases aligned with CIN 1, while HSIL and AGUS were associated with higher-grade lesions (CIN 2/3). These results confirm that both VIA and Pap smear are effective screening tools that align well with colposcopic evaluation.

**Table 4:** Association of VIA and Pap outcomes with colposcopy results (n=58)* *Data presented as number (%) p<0.05: statistically significant χ2: chi-squared test, VIA: visual inspection of acetic acid, Pap: Papanicolaou smear, AGUS: atypical glandular cells of undetermined significance, ASCUS: atypical squamous cells of undetermined significance, HSIL: high-grade squamous intraepithelial lesion, LSIL: low-grade squamous intraepithelial lesion, NILM: negative for intraepithelial lesion or malignancy, CIN: cervical intraepithelial neoplasia

Parameters		Colposcopy results					χ^2^ value	p-value
		Chronic cervicitis	CIN 1	CIN 2	CIN 3	Normal		
VIA results	Negative	0 (0)	0 (0)	0 (0)	0 (0)	2 (3.4)	37.976	<0.0001
	Positive	17 (29.3)	28 (48.3)	5 (8.6)	5 (8.6)	1 (1.7)		
Pap results	AGUS	0 (0)	0 (0)	0 (0)	2 (3.4)	0 (0)	100.618	<0.0001
	ASCUS	1 (1.7)	21 (36.2)	1 (1.7)	0 (0)	1 (1.7)		
	HSIL	0 (0)	0 (0)	1 (1.6)	3 (5.2)	0 (0)		
	LSIL	0 (0)	5 (8.6)	3 (5.2)	0 (0)	1 (1.7)		
	NILM	16 (27.6)	2 (3.4)	0 (0)	0 (0)	1 (1.7)		

There was a statistically significant association between colposcopy and histopathological findings (χ²=10.000, p=0.007), demonstrating that colposcopy is highly effective in detecting cervical lesions when validated against histopathology, the diagnostic gold standard (Table 5). This was further supported by a very strong positive correlation between colposcopic and histopathological grading (Pearson's R=0.905, p<0.0001), indicating a high degree of agreement in lesion severity assessment.

**Table 5:** Association of colposcopy results with HPE results (n=10)* *Data presented as number (%) p<0.05: statistically significant χ2: chi-squared test, HPE: histopathological examination, CIN: cervical intraepithelial neoplasia, SCC: squamous cell carcinoma

Parameters		Colposcopy results		χ^2^ value	p-value
		CIN 2	CIN 3		
HPE results	CIN 2	5 (50)	0 (0)	10.000	0.007
	CIN 3	0 (0)	4 (40)		
	SCC	0 (0)	1 (10)		

VIA shows high sensitivity but very low specificity, indicating a high false-positive rate. It is useful as a broad screening tool, but it must be followed by colposcopy for confirmation. The Pap smear demonstrates high sensitivity and specificity, with excellent positive predictive value (PPV) and negative predictive value (NPV), indicating that it is more accurate and reliable for detecting true cases (Table 6).

**Table 6:** Sensitivity, specificity, PPV, NPV, and accuracy of VIA and Pap compared to colposcopy (n=58) VIA: visual inspection of acetic acid, Pap: Papanicolaou smear, PPV: positive predictive value, NPV: negative predictive value

Diagnostic test performance	VIA	Pap
Sensitivity	100%	94.7%
Specificity	10%	85%
PPV	67.9%	92.3%
NPV	100%	89.5%
Accuracy	68.9%	91.4%

Significant associations, as determined by the chi-squared test, were observed between abnormal VIA and Pap smear results with early age at marriage between 18 and 20 years (p=0.002 and p=0.008, respectively), lower socioeconomic status (p=0.001 and p=0.024, respectively), and high parity (p<0.0001 and p=0.002, respectively) (Table 7). No statistically significant association was found between screening outcomes and different age groups or rural/urban livelihood (Table 7).

**Table 7:** Association of abnormal VIA and Pap smear outcomes with demographic parameters (N=170)* *Data presented as number (%) p<0.05: statistically significant VIA: visual inspection of acetic acid, Pap: Papanicolaou smear, SES: socioeconomic status, χ2: chi-squared test

Demographic parameters		VIA				Pap smear			
		Negative (n=114)	Positive (n=56)	χ^2^ value	p-value	Abnormal (n=39)	Normal (n=131)	χ^2^ value	p-value
Age group (years)	25-35	45 (26.5)	14 (8.2)	5.334	0.149	12 (7.1)	47 (27.7)	1.057	0.787
	36-45	35 (20.6)	19 (11.2)			13 (7.6)	41 (24.1)		
	46-55	32 (18.8)	23 (13.5)			14 (8.2)	41 (24.1)		
	56-65	2 (1.2)	0 (0)			0 (0)	2 (1.2)		
Age at marriage (years)	18-20	42 (24.7)	35 (20.6)	12.160	0.002	24 (14.1)	53 (31.2)	9.552	0.008
	21-24	69 (40.6)	18 (10.5)			12 (7)	75 (44.1)		
	≥25	3 (1.8)	3 (1.8)			3 (1.8)	3 (1.8)		
SES	Upper	6 (3.5)	10 (5.9)	18.137	0.001	6 (3.5)	10 (5.9)	11.203	0.024
	Upper middle	12 (7.1)	7 (4.1)			7 (4.1)	12 (7.1)		
	Lower middle	4 (2.4)	9 (5.3)			6 (3.5)	7 (4.1)		
	Upper lower	24 (14.1)	7 (4.1)			4 (2.4)	27 (15.9)		
	Lower	68 (40)	23 (13.5)			16 (9.4)	75 (44.1)		
Livelihood	Rural	93 (54.7)	47 (27.6)	0.143	0.706	29 (17)	111 (65.3)	2.225	0.136
	Urban	21 (12.4)	9 (5.3)			10 (5.9)	20 (11.8)		
Parity	1	33 (19.5)	6 (3.5)	25.855	<0.0001	3 (1.8)	36 (21.2)	15.053	0.002
	2	49 (28.8)	19 (11.2)			14 (8.2)	54 (31.8)		
	3	31 (18.2)	20 (11.8)			15 (8.8)	36 (21.2)		
	≥4	1 (0.6)	11 (6.4)			7 (4.1)	5 (2.9)		

## Discussion

Cervical cancer remains a significant public health challenge, especially in low- and middle-income countries (LMICs) like India, where it constitutes one of the leading causes of cancer-related deaths among women, although it can be largely prevented with early detection and treatment of precancerous lesions [3,4]. This study aimed to assess the effectiveness of the Pap and VIA as primary screening tools and to evaluate their correlation with colposcopic findings in detecting cervical lesions.

Symptomatology echoed broader trends, with vaginal discharge as the most common complaint in 155/170 (91.2%), a rate comparable to two different studies by Khan et al. [20] and Sachan et al. [23], who reported a similar result. This underscores that many women in resource-constrained settings often seek care only after developing symptoms and present with advanced or symptomatic lesions, reducing opportunities for early intervention.

The sensitivity and specificity values obtained for VIA (100% and 10%, respectively) align with previous literature, indicating that VIA, although highly sensitive, tends to have low specificity due to a high rate of false positives [13,19]. These limitations were also evident in our results, where a significant number of VIA-positive cases were later diagnosed with benign findings. The observed 100% sensitivity and negative predictive value (NPV) of VIA in this study may reflect overestimation due to the small number of biopsy-confirmed cases and reliance on colposcopy rather than histopathology as the reference standard. The utility of VIA lies in its feasibility for mass screening, particularly in resource-limited settings. A meta-analysis by Viñals et al. emphasized the advantages of VIA for opportunistic screening in LMICs, although it also noted its variable accuracy depending on the examiner's expertise [13]. Buchade et al.'s study advocated for the use of VIA as a large-scale screening approach and as a screening modality, particularly in settings with limited resources [24]. A systematic review and meta-analysis by Lohiya et al. concluded that while VIA screening may reduce cervical cancer mortality within a decade, longer follow-up is needed to impact incidence, underscoring the value of scaling up VIA in resource-poor settings [25].

Pap smear, on the other hand, demonstrated a sensitivity of 94.7% and specificity of 85%, with high positive predictive value (PPV) and negative predictive value (NPV), confirming its reliability in detecting true cases of cervical intraepithelial neoplasia. These findings reinforce its value as the standard of excellence for cytological screening, where laboratory infrastructure and trained cytotechnicians are available. Comparable results were noted by Kaya Terzi et al., who reported improved diagnostic accuracy when Pap smears were coupled with HPV testing [12]. A systematic review and meta-analysis by Smith et al. reported that primary HPV testing had the highest sensitivity (79.5%) and good specificity (72.6%) for detecting CIN 2+, while VIA showed 72.3% sensitivity and 74.5% specificity, and Pap smear demonstrated 60.2% sensitivity with the highest specificity at 97.4% [26]. Our findings reinforce the critical role of VIA and Pap smear as frontline tools in cervical cancer screening.

The results from our study underscore colposcopy's role as a definitive diagnostic modality, particularly when used to guide biopsy of suspicious lesions. Cooper et al. observed similar findings, highlighting the utility of colposcopy in targeted evaluation and biopsy for women with abnormal cytology or VIA findings [14].

The association between VIA and colposcopic results (p<0.0001), as well as Pap smear and colposcopic findings (p<0.0001), supports the complementary nature of these screening methods. When used together, they enhance the detection of precancerous lesions, a finding echoed by Khan et al., who advocated the combined use of VIA and Pap smear as a cost-effective and efficient dual strategy in settings with constrained resources [20].

The most common histopathological diagnoses among the 10 biopsied included CIN 2 in 5/10 (50%) and CIN 3 in 4/10 (40%), and only 1/10 (10%) was diagnosed as squamous cell carcinoma (SCC), reflecting the benefit of early detection through screening. These results corroborate findings by Höhn et al., who emphasized the importance of accurate histological classification based on the updated WHO guidelines for female genital tract tumors [22].

The demographic profile of participants reveals important associations. Women married before the age of 20, those with higher parity (≥3), and women from lower socioeconomic strata showed increased rates of VIA and Pap smear positivity with significant associations. This observation is consistent with earlier reports by Tekalegn et al. [6], Mekonnen et al. [7], and Nessa et al. [27], who linked early sexual debut, multiparity, and poor socioeconomic conditions to higher risks of cervical cancer. The higher VIA positivity observed among women from lower socioeconomic backgrounds may partially reflect false positives due to inflammatory cervical changes commonly associated with poor hygiene, recurrent infections, or high parity in these populations.

Our study's findings also highlight the ongoing challenges in rural populations. While no statistically significant difference was found between rural and urban women in terms of test positivity, a majority (140/170 (82.4%)) of our participants resided in rural areas, emphasizing the need for targeted outreach in these regions. Barriers such as limited awareness, stigma, and lack of access to services often impede cervical cancer screening uptake in rural LMIC settings [15,28].

Although Pap smear and colposcopy are effective, their implementation in rural and resource-constrained settings is limited by infrastructure requirements, the need for trained personnel, and cost. VIA, being a low-cost, easily administered screening test with immediate results, emerges as a practical alternative, especially when integrated with community health programs. Recent advances in AI-assisted VIA interpretation may further reduce the dependency on clinical expertise, improving its utility in field conditions [13]. Although not assessed in this study, AI-assisted VIA may help reduce interobserver variability and enhance diagnostic accuracy, especially in nurse-led, task-shifted screening programs in low-resource settings.

The WHO has set a global strategy to eliminate cervical cancer as a public health problem by the year 2030 [29]. This goal is defined as achieving an incidence rate of less than four cases per 100,000 women per year [29]. To achieve this goal, the WHO has proposed the "90-70-90" strategy: vaccinating 90% of girls against HPV by age 15, screening 70% of women with a high-performance test (such as HPV DNA testing) at ages 35 and 45, and ensuring that 90% of women diagnosed with cervical disease receive timely and appropriate treatment [29].

Despite national guidelines, major gaps persist in cervical cancer screening implementation across LMICs, including limited community awareness, inadequate follow-up, and workforce shortages. Scalable solutions such as mobile VIA camps, digital patient tracking systems, and decentralized training for mid-level health providers are urgently needed to close these gaps and improve program coverage.

The strength of this study lies in its prospective design, well-defined inclusion and exclusion criteria, and the use of a combination of screening modalities in the real-world, low-resource setting in a semi-urban Indian population with limited access to advanced diagnostics.

This study also has several limitations. This single-center, hospital-based study included mostly symptomatic women, which may introduce selection bias and limit generalizability. The small number of colposcopy-guided biopsies (n=10) and reliance on colposcopy rather than histopathology for most analyses raise the risk of verification bias and may overestimate VIA sensitivity and NPV. HPV DNA testing, a gold standard screening method, was not performed due to resource constraints, limiting comparative assessment. VIA's examiner-dependent nature, absence of interobserver variability assessment, and lack of quality assurance measures may have influenced specificity. The study also did not perform multivariate analysis to adjust for confounders, and long-term follow-up data were unavailable to assess lesion outcomes. Future multicenter, longitudinal studies incorporating HPV testing and regression analysis are recommended to validate and strengthen these findings.

## Conclusions

In conclusion, this study reaffirms the importance of combining VIA and Pap smear as complementary tools for the early detection of cervical neoplasia, with colposcopy serving as a vital confirmatory diagnostic step. VIA offers a feasible and scalable approach for mass screening in underserved settings, while Pap smear adds specificity and reliability where feasible. A tiered screening model should be implemented, wherein VIA is conducted at the primary care level by trained frontline workers, with positive cases referred for Pap smear and colposcopy at secondary or tertiary centers. The integration of these methods into national screening programs, especially in LMICs, can significantly improve early detection, reduce the burden of cervical cancer, and ultimately save lives. However, the findings should be interpreted with caution, given the small number of biopsy-confirmed cases and the absence of HPV testing. Future efforts should focus on strengthening health infrastructure, enhancing training for frontline health workers, promoting HPV vaccination, and incorporating molecular testing. Longitudinal and multicenter studies are needed to validate these findings and guide implementation at scale. Practical recommendations include incorporating VIA into primary-level services, establishing clear referral and tracking mechanisms, and ensuring quality assurance at all stages of the screening pathway to align with the WHO's 2030 elimination targets.
